# Supplementary material for: Smooth Interpolating Curves with Local Control and Monotone Alternating Curvature
Source: Comput Graph Forum. 2022 Oct 6;41(5):25–38. doi: 10.1111/cgf.14600 (PMC9827861; doi:10.1111/cgf.14600)
Supplement: Supplementary file 1 — Supplement Material [file CGF-41-25-s001.zip › Local-Smooth-Interpolating-MonoCurvature/extern/clothoids/submodules/Utils/docs/api-cpp/program_listing_file_Utils_zstr.hh.html]

Program Listing for File Utils\_zstr.hh — Utils v1.2

### Navigation

- index
- toc
- Utils »
- Program Listing for File Utils\_zstr.hh

# Program Listing for File Utils\_zstr.hh¶

↰ Return to documentation for file (`Utils_zstr.hh`)

```
/*--------------------------------------------------------------------------*\
 |                                                                          |
 |  Copyright (C) 2017                                                      |
 |                                                                          |
 |         , __                 , __                                        |
 |        /|/  \               /|/  \                                       |
 |         | __/ _   ,_         | __/ _   ,_                                |
 |         |   \|/  /  |  |   | |   \|/  /  |  |   |                        |
 |         |(__/|__/   |_/ \_/|/|(__/|__/   |_/ \_/|/                       |
 |                           /|                   /|                        |
 |                           \|                   \|                        |
 |                                                                          |
 |      Enrico Bertolazzi                                                   |
 |      Dipartimento di Ingegneria Industriale                              |
 |      Universita` degli Studi di Trento                                   |
 |      email: enrico.bertolazzi@unitn.it                                   |
 |                                                                          |
\*--------------------------------------------------------------------------*/

#pragma once

#ifndef DOXYGEN_SHOULD_SKIP_THIS

#ifndef UTILS_ZSTREAM_dot_HH
#define UTILS_ZSTREAM_dot_HH

#ifdef __clang__
#pragma clang diagnostic push
#pragma clang diagnostic ignored "-Wc++98-compat"
#pragma clang diagnostic ignored "-Wc++98-compat-pedantic"
#pragma clang diagnostic ignored "-Wold-style-cast"
#pragma clang diagnostic ignored "-Wswitch-enum"
#pragma clang diagnostic ignored "-Wdocumentation"
#pragma clang diagnostic ignored "-Wdocumentation-unknown-command"
#pragma clang diagnostic ignored "-Wglobal-constructors"
#pragma clang diagnostic ignored "-Wzero-as-null-pointer-constant"
#pragma clang diagnostic ignored "-Wweak-vtables"
#pragma clang diagnostic ignored "-Wshorten-64-to-32"
#pragma clang diagnostic ignored "-Wundefined-func-template"
#pragma clang diagnostic ignored "-Wdouble-promotion"
#pragma clang diagnostic ignored "-Wsigned-enum-bitfield"
#pragma clang diagnostic ignored "-Wsign-conversion"
#pragma clang diagnostic ignored "-Wweak-vtables"
#pragma clang diagnostic ignored "-Wunused-template"
#pragma clang diagnostic ignored "-Wnon-virtual-dtor"
#pragma clang diagnostic ignored "-Wpadded"
#endif

#include "Utils/Utils.hxx"

#include "Utils/zstr/zstr.hpp"

#ifdef __clang__
#pragma clang diagnostic pop
#endif

#endif

#endif
```

### Quick search

### Table of Contents

- rang - Colors for your Terminal
- Zstr
- C++ API

«
hide menu

menu
sidebar
»

### Navigation

- index
- toc
- Utils »
- Program Listing for File Utils\_zstr.hh

© Copyright 2021, Enrico Bertolazzi.
Created using Sphinx 4.2.0.
